# Smoke on the horizon: leveling up citizen and social science to motivate health protective responses during wildfires

**DOI:** 10.1057/s41599-024-02641-1

**Published:** 2024-02-12

**Authors:** Steven E. Prince, Sarah E. Muskin, Samantha J. Kramer, ShihMing Huang, Timothy Blakey, Ana G. Rappold

**Affiliations:** 1 Center for Public Health and Environmental Assessment, Office of Research and Development, United States Environmental Protection Agency, Research Triangle Park, NC, USA; 2 Oak Ridge Institute for Science and Education at the Center for Public Health and Environmental Assessment, Office of Research and Development, United States Environmental Protection Agency, Research Triangle Park, NC, USA; 3 Sonoma Technology Incorporated, 1450 N, McDowell Blvd., Suite 200, Petaluma, CA, USA

## Abstract

Climate change factors and expanded population growth in the Wildland Urban Interface (transition zone between human structures and undeveloped wildland) contribute to a projected increase in wildfire frequency and smoke exposure. As an unregulated source of air pollution, reducing smoke exposure represents a difficult challenge for health risk communicators. The target audience is broad with unpredictable health impacts due to spatial and temporal variability in exposure. Beyond providing information, agencies face challenges reaching affected populations, motivating behavior change, and overcoming barriers between intentions and actions (recommended health protection). The Smoke Sense citizen science project developed a smartphone app to provide an engagement, learning, and information-sharing platform. Here we draw upon previous trends in behavioral patterns and propose a synergistic approach of citizen and behavioral science that can be applied to increase understanding of health risk and motivate new habits to reduce exposure among impacted individuals. Presentation of the approach proceeds as follows: (1) we identify several core factors that contribute to an intention-action gap, (2) identify applicable social and behavioral science principles that can bridge the gap, (3) propose explicit examples focused on theoretical principles, (4) describe small-scale user preliminary feedback and examples for monitoring and evaluating impact, and (5) provide a look to the future for collaborative citizen engagement. Current health risk communication strategies often lack consideration of behavioral factors that may enhance motivation and encourage behavior change. The proposed approach aims to leverage the strengths of citizen and social science and seeks to encourage a focused ‘digital community’ to implement new habits in the face of unpredictable and dynamic environmental threats.

## Introduction

Increased particulate matter pollution, especially related to wildfire smoke exposure, has been linked to a broad range of health outcomes including higher rates of cardiorespiratory emergency visits, hospitalizations, and even death (Adetona et al., 2016; Black et al., 2017; Dennekamp and Abramson, 2011; Dodd et al., 2018; Haikerwal et al., 2015; Liu et al., 2015; Morgan et al., 2010; Rappold et al., 2011; Reid et al., 2016; Wettstein et al., 2018), kidney (Wyatt et al., 2020) and cognitive (Cleland et al., 2022) impacts. As the risks from large wildfires grow on the landscape with hotter and drier climate conditions, a directly related public health danger looms on the horizon in the form of greater and more frequent wildfire smoke exposure. Smoke exposure extends well beyond fire perimeters and jurisdictional boundaries and results in billions of dollars in health burden (Fann et al., 2018; Jones, 2017; Kochi et al., 2010). Current approaches to mitigation of wildfire smoke health risks are projected to have ‘modest and unequal benefits’ (Burke et al. 2022). Wildfire smoke exposure has a range of impacts; from those that seem minor or nuisances such as upper respiratory, ear-nose-throat symptoms, or scratchy eyes to headaches, depression, anxiety, and impaired sleep to the most extreme outcomes previously described. The heterogeneity of effects on just respiratory health alone makes it difficult to characterize demographic subgroups that are more susceptible to adverse outcomes (Kondo et al., 2019). Simply stated, no one in the vicinity of a fire; young or old, healthy or in poor health, can completely escape the ramifications of smoke exposure including an increasing public health burden.

Frequent wildfire smoke from events near or far have made fire season an everyday life consideration in some communities, while others are just beginning to experience impacts. Significant population growth has occurred in recent decades along the Wildland-Urban Interface (WUI; the zone where human structures and wildland intersect), exacerbating the risk of fire and smoke exposure (Peterson et al., 2021). These trends highlight the need to identify health risk communication approaches that better motivate individuals to change behavior and adopt health risk mitigation strategies. Although recommended health responses focus on reducing the amount and time of smoke exposure, audiences may need additional context to move from intentions to actions, especially if those actions are perceived as difficult, costly, or more of a change than they are initially willing to consider.

In this manuscript, we use the Smoke Sense citizen science project as a platform to demonstrate and test how social science concepts can be used to facilitate motivation, engagement, and provide efficacy to users contemplating protective action. The goal is to contribute to efforts to reduce the public health burden by closing the gap between intentions and actions that occurs when experiencing smoke events. Specifically, we draw upon the behaviors and experiences observed among participants who have experienced smoke exposure in previous years. We describe an approach to reduce barriers (friction) that slow or prevent people from implementing protective exposure reducing behaviors. Similar approaches have been used in behavioral medicine, for example to design interventions to improve exercise adherence and outcomes in cardiac rehabilitation programs (Sniehotta et al., 2005) or healthy behaviors that facilitate weight loss maintenance (Asbjornsen et al., 2022; Asbjornsen et al., 2020).

Methods proposed here are rooted in established social and behavioral science theory but have not been linked to behavioral patterns during smoke exposure episodes. For each concept, we identify specific factors contributing to the intention-action gap and connect them to a relevant theoretical framework.

### Approach.

Smoke Sense is a citizen science project, launched by researchers at the U.S. Environmental Protection Agency, that has both investigative and educational objectives as an interactive platform for building knowledge about wildfire smoke, health, and protective actions. The investigative objective of Smoke Sense is to reach individuals when and where they are affected by smoke and learn the context within which health risk communications are delivered. The educational objective is to increase awareness and deliver real-time information to and between participants. Participants engage with a smartphone application to explore current and forecast visualizations of air quality, learn about how to protect health from wildfire smoke, and share their smoke experiences, health symptoms, and behaviors taken to reduce their exposures to smoke with the wider group of users. This citizen science platform provides timely and relevant information to those affected by wildfire smoke exposure (the user community) while also allowing them to share perceptions and actions related to air quality more generally. For some this may involve reducing fear and uncertainty, while for others, it may involve reminders to stay vigilant and guard against complacency. In all cases, individual users can learn and interact with air quality and other citizen scientists. As such, learning about the issue of wildfire smoke and health advances knowledge at the level of individual participants but also overall, across participants.

Smoke Sense has employed an interactive and growing process of learning. With inputs across >60,000 participants as well as a wider community of stakeholders, Smoke Sense has provided insights about how current health protective recommendations are perceived, adopted, and adhered to by individuals impacted by smoke (Hano et al., 2020a; Hano et al., 2020b; Postma et al., 2022; Rappold et al., 2019). The insights learned identified gaps that need to be bridged for health risk communication to better motivate people to adopt health risk measures in a timely manner. Table 1 demonstrates the concept where citizen science can increase engagement and boost motivation for action. Text boxes define terms and also list some potential barriers, hypothetical questions, and mindsets that might contribute to inaction or delayed health protective action.

### Why does the intention-action gap occur in the context of smoke exposure? A brief history and the current state of smoke response.

Specific health behavior guidelines that are recommended during smoke events go unacted upon for many reasons. Fire and smoke are often unpredictable, arising unexpectedly with uncertainty in the duration and locations of impact, yet requiring an urgency to act in response. Biases and human nature contribute to an individual’s response to wildfire smoke air pollution and although recommended protective actions should benefit health outcomes, they are unlikely to be the default behavioral response for an “average” individual (Santana et al., 2021). Simply put, maintaining the status quo is an easier response than changing behaviors. The common perception that risk is higher for those with respiratory issues (Mirabelli et al., 2018) may be interpreted as a reduced need for concern by “healthy” individuals (Hano et al., 2020a). Similarly, messages that reinforce the increased susceptibility of children, the elderly, and pregnant women to the health effects of pollution may unintentionally serve to downplay perceived risk for populations who are not explicitly referenced by such messages. Recent papers suggest that larger segments of the population than commonly conceived are indeed subject to exposure and health risks (Burke et al., 2022; Rappold et al., 2017).

Wildfire impacts on air quality are challenging to predict, compounding the issue of variable risk perceptions associated with specific populations or pre-existing medical conditions. Whereas threats to life and property associated with fire often prompt warnings to take protective actions and elicit immediate concern such as evacuation, risks from smoke can be perceived as slower moving, less urgent, and more remote in distance. This dynamic can contribute to dramatic air quality changes driven by smoke being relegated to the status of merely a troublesome side-effect of the “headline-grabbing” threat. Although the magnitude of wildfire impacts (economic impacts, recreation, tourism, etc.) and greater proximity of human habitation to wildland areas (Wildland Urban Interface) have contributed to greater issue awareness, unpredictability and uncertainties associated with wildfire smoke exposure can leave people with decision paralysis. Other barriers to action include mental, physical, and economic costs associated with changing behavior in response to a transient event. In the absence of feelings of efficacy for behavioral responses to improve an outcome, a status quo bias or “wait-and-see” approach is more likely as a default response.

In addition to biases that contribute to a lack of response inertia, some protective behaviors for avoiding smoke exposure occur outside of public view. Private actions make it difficult to assess how common or uncommon it is to take protective action. For example, attenuating indoor infiltration (closing doors and windows), filtering the air to reduce pollution concentration (using a portable HEPA filter and/ or air conditioning with filtration), and not engaging in behaviors that add indoor pollution (frying foods, using candles or incense, smoking, vacuuming with a non-HEPA device) typically all occur in private dwellings. Sales of commercial HEPA filters and filter media may signal desired action, but it is unclear how often these devices are used, despite studies indicating their potential to reduce indoor exposure to wildfire smoke (Barn et al., 2008; Fisk and Chan, 2017; Mott et al., 2002; Xiang et al., 2021). When stores sell out of such protective tools during the most extreme smoke events as customers flock to buy them, we can presume it is because action was deemed necessary. In these cases, inertia to act is observable but with the unfortunate consequence of leaving many of those who are seeking protective interventions unable to act on their intentions. The prevalence of behaviors that pertain to the outdoors, such as recommendations to reduce strenuous outdoor activity or time spent outdoors during high pollution, is also difficult to assess. In recent years, social media has successfully made private behaviors ‘visible’ (diet, exercise, meditation) increasing the encounters with new habits that individuals and communities seek to develop.

A notable exception to response actions that happen in private is the use of N95 respirators or protective facemasks. During several recent U.S. wildfire episodes with extreme smoke impacts, hardware stores began selling out of N95 respirators, with news reports covering the “frenzied” rush of customers trying to find and purchase any existing stock (Dianne de Guzman and SFGATE, 2018; News, 2018). In this case, media coverage revealed the common desire to mitigate exposure, which could then be reinforced by seeing others buying and using respirators. The timing of rushing to take protective actions is likely to lag the precipitating conditions as people accumulate information about the increasing commonality of such behaviors. In circumstances like this, the user community may be able to ‘see’ the commonality of interventions earlier in the time course of a smoke event. A related goal to motivating action is to guide the user community to beneficial times and suitable instruction (Chen et al., 2022a; Chen et al., 2022b; Prince et al., 2021) for using an intervention, which is likely prior to, as well as during the most extreme conditions.

Finally, people are likely to be influenced to a greater extent by those who are nearby and share similar sociodemographic characteristics (Dressler et al., 2017). Protective behaviors have costs (both real and perceived) that impact their presumed and actual adoption. Together, these factors contribute to uncertainty about behavior change in the context of extreme pollution and increase the likelihood of doing nothing or waiting until the impacts are even more severe (Rappold et al., 2019). To meaningfully address the needs of increasing numbers of people who will experience smoke exposure, we hypothesize that general principles from social science can anticipate sources of behavioral friction and provide a suite of solutions. More specifically, we hypothesize that the use of social norms, positive reinforcement, reducing friction, and increasing self-efficacy can empower timely action and health protective patterns of response.

### Leveraging social science to motivate behavior change.

Although health behavior guidelines are available, there is nonetheless a gap in adopting protective behaviors to reduce air pollution exposure. Most of the communication and education so far has been focused on providing information. Information is an important tool for highly motivated individuals, but providing information alone is less likely to sustain long-term behavior change. Providing the right message at the right time is part of a broader set of strategies to foster behavioral change. Here we provide a link between the gaps and barriers to action and the planned implementation of a social science approach in the context of the Smoke Sense app.

Based on insights into common behavioral patterns observed from past Smoke Sense participants, we use pillars of behavioral and social science relevant to risk perception and habit formation to address the goal of enhanced motivation and establishment of new air quality habits (Slovic, 1987; Tversky and Kahneman, 1974, 1981). More specifically, we link the gaps in behavioral adoption to four social science domains that can be tested in a future implementation of the app by introducing features to address social norming, positive reinforcement, reducing friction, and enhancing self-efficacy which are defined and described below. We provide specific examples and features that can be implemented in the Smoke Sense app to reach the objectives. A citizen science project is an opportunity to test the effectiveness of previously recognized social science strategies to motivate behavior change in the context of environmental health hazards.

### Social norms.

Social norms are commonly defined as informal rules or standards that govern, guide, or constrain behavior in groups or societies (Bicchieri et al., 2018; Cialdini and Trost, 1998; Dempsey et al., 2018). Social norms do not operate in isolation, but rather contribute to a suite of behavioral drivers that include amongst other factors, attitudes, beliefs, habits, and prior behaviors. While social norms do not have the force of law, they can relate to a perceived social pressure to engage or not engage in specific behaviors (Ajzen, 1991, 2001). A further distinction is made between descriptive norms, or the observed levels of a given behavior, and injunctive norms, or the perceived appropriateness of a given behavior (what the correct course of action ought to be). While established social norms are thought of as static (less prone to change), so-called dynamic norms can be used to characterize increasing acceptance in cases when a behavior is less common.

Recent studies have investigated whether information about the direction of collective change might serve as a nudge to behaviors, especially in the context of sustainability. Less common behaviors can be described in terms of their recent change, which might be interpreted as representing a shift in the established norm towards the direction of a new social norm. Examples include reducing consumption of meat or increasing use of reusable cups versus disposable cups (Loschelder et al., 2019; Sparkman and Walton, 2017). Given the relative novelty of wildfire smoke exposure as a public health concern and the difficulties in gauging societal concern and appropriate response levels, social norms are to some extent being constructed with each subsequent smoke episode. Each smoke event provides opportunities for learning about the potential for repeated exposure and the need to be prepared to act in impactful ways regardless of a person’s familiarity with the topic beforehand. We hypothesize that providing an app with the capability to view and share health-protective action data could help to nudge impacted communities, establish or change norms, and develop new habits related to air pollution.

**Fig. 1:**
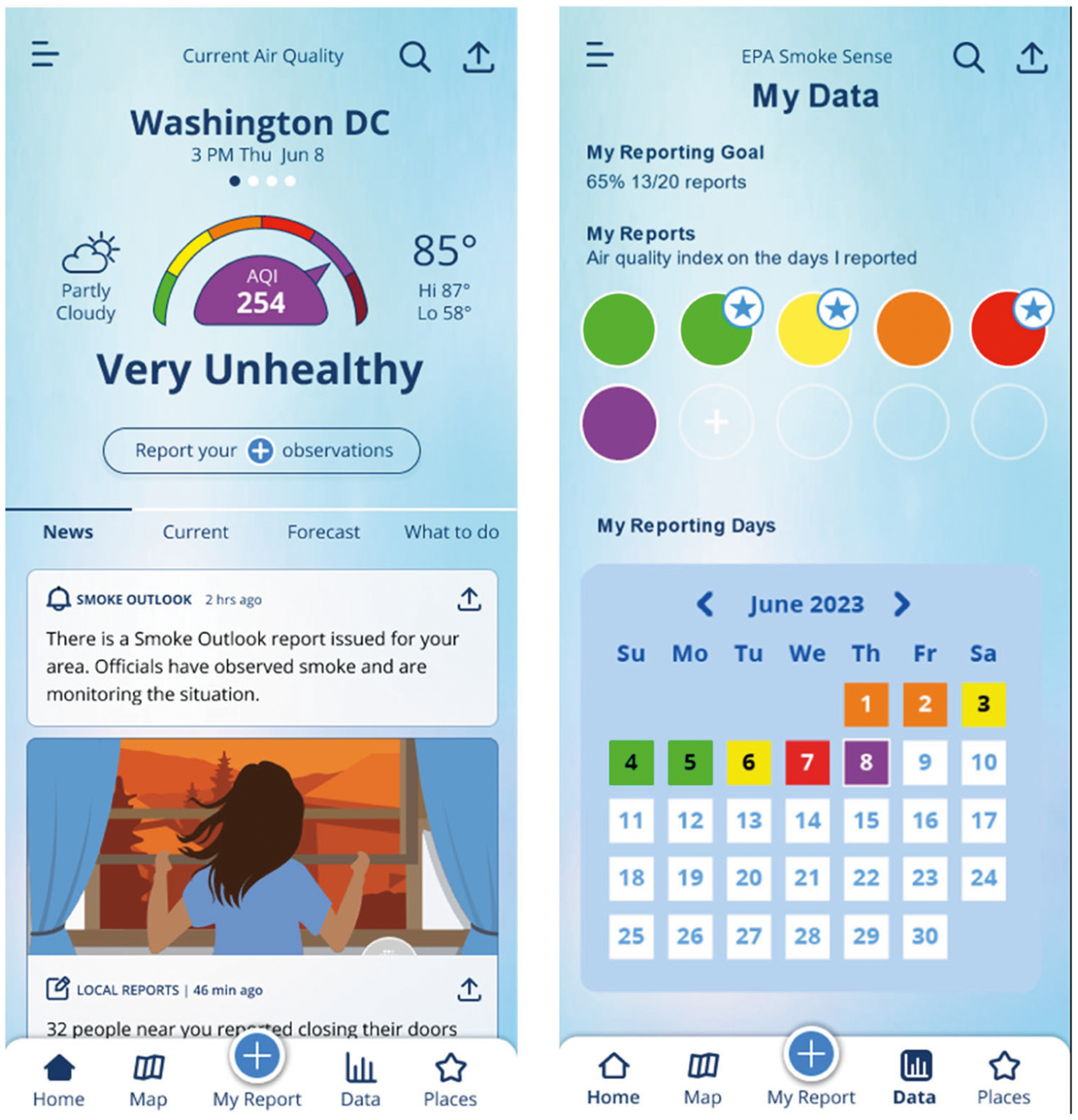
Home screen (left) and My Data module (right). A ‘newsfeed’ could provide information characterizing recent responses to local air quality conditions. The My Data module would provide a report summary and calendar view for personal reporting and air quality history experienced. Over time, participants should become more familiar with how their behavior compares to a social norm.

In the context of Smoke Sense or other app-based interactions, references to social norms can be implemented in several ways. For example, a “newsfeed” could be utilized to deliver messages responsive to current and changing conditions displaying current social norms. In Smoke Sense, a newsfeed function can be embedded on the Dashboard and populated with air quality information and educational features, with a focus to share aggregated data from user reports to help establish social norms around behaviors that protect from wildfire smoke exposure (Fig. 1). For example, a block of the newsfeed might read: “74 people in your zip code have used room HEPA filters in the past 24 h.” In this case, we hypothesize the newsfeed can change both the descriptive and the injunctive norm for HEPA filter behavior, and prod users to consider changing their own habits and adopting that same behavior (Curtis et al., 2009).

Dynamic norms can also be used to highlight behaviors that are becoming more common, while not yet achieving widespread adoption. Using the map module to display similar information can allow users to visualize behavior in a graphical format and see how responses track in relation to the spatial distribution of smoke and air quality impacts. Finally, the ability to view your own data and how you respond to various pollution threats facilitates comparison with map and newsfeed aggregated data of nearby users and the broader population of users (Fig. 1). Such comparisons can also be explicitly tied to user data as an example of social proof, indicating to users the behaviors for which they respond similarly to the group or other actions where they might be able to improve.

These tools have a goal to broaden the tendency to share dramatic impacts of environmental air quality changes, such as “unearthly” colors in the sky or severely reduced visibility. Sharing of the types of preparation and responses associated with high pollution episodes helps to validate the commonality and appropriateness of protective actions. Such sharing not only influences perceptions of social norms but can enhance personal efficacy related to the rationale for considering and implementing protective actions. An excuse that nobody else shows concern or acts to reduce exposure is a potential barrier that can also be reduced. Finally, positive language used to provide information and feedback to users can provide social reinforcement as users become more familiar with a suite of appropriate behavioral responses.

### Positive + social reinforcement.

Positive reinforcement relies on rewards or incentives that occur after a desired behavior. The reward reinforces the behavior, making the behavior more likely to reoccur. While rewards are often tangible (money, gift cards, treats) or tokens (points, stickers), they can also be nonfinancial, including natural reinforcers (getting good grades after studying) and social reinforcers (verbal praise, acknowledgement, and approval of behaviors, especially with peer awareness). In the case of air quality and Smoke Sense, we seek to reinforce both protective health behaviors, and general use of the app. More frequent use of the app should increase a user’s understanding and awareness of air quality and their agency for reducing exposure to smoke and related health impacts. Rewards need to be salient both for behaviors that increase protection from smoke exposure, and behaviors that enhance awareness and understanding of air quality information. Learning about smoke pollution and protective actions requires an initial (shorter term) motivation to seek information and understanding. However, maintaining motivation to engage with the issue over a longer period of time requires more sustained reinforcement.

**Fig. 2:**
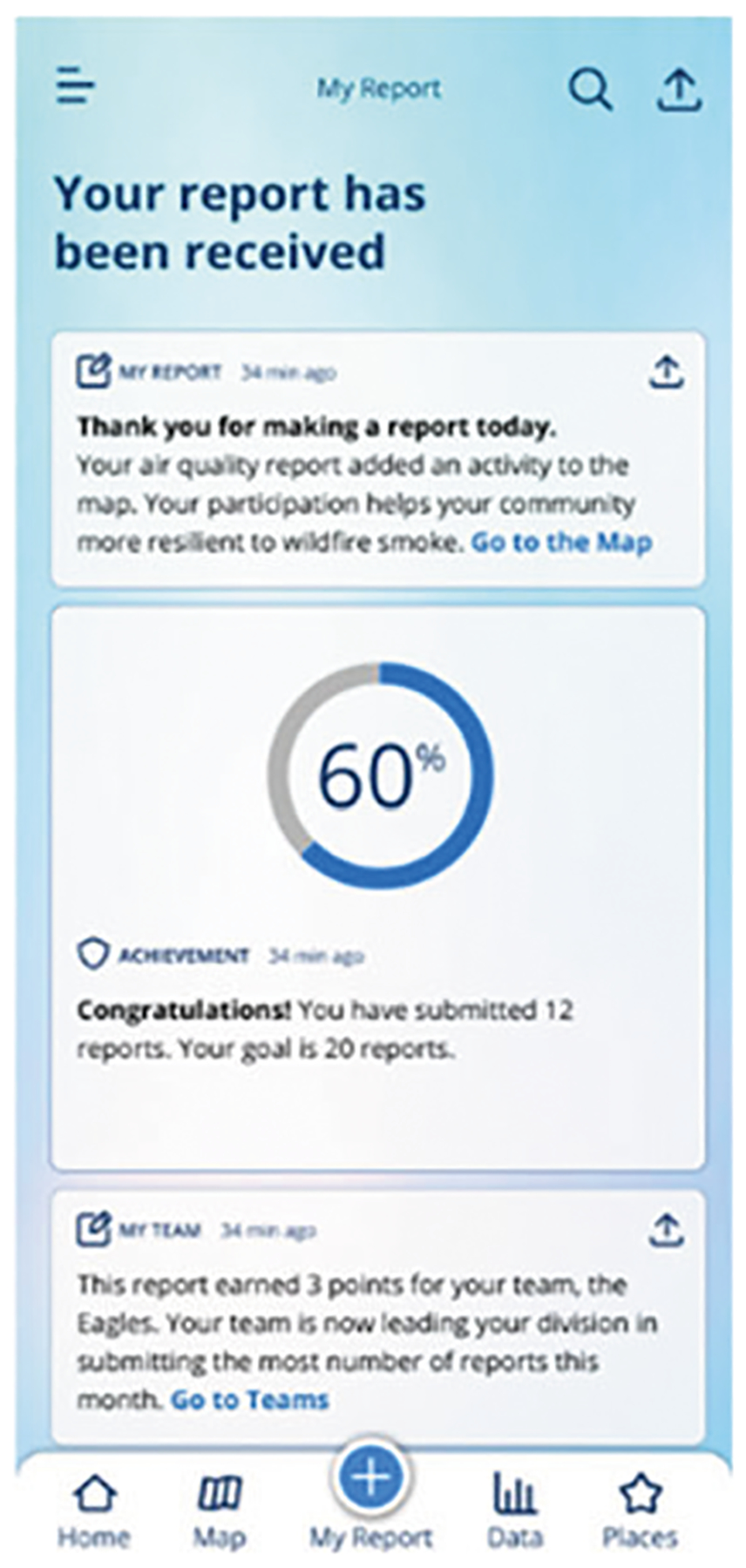
Report received acknowledgment. Immediately following report submission, messages would acknowledge participant contribution, describing the benefit to the community, progress to a personal goal, and points earned. Easy access to viewing the map and is available to provide instant updating.

The app-based communication platform can incorporate features that immediately reinforce or reward users with feedback, such as praise-based acknowledgment of a report submission, earning points for submitting or sharing a report, and alerting that their report has been shared to the map (the peer community) (Fig. 2). Sustained reinforcement features include: the ability to review ‘stars’ and points received in calendar and user data synopsis formats associated with taking appropriate air quality actions; praise for achieving a preset number of reports (goal); privileges related to demonstrating gains in knowledge and awareness; and weighing in on the selection of features under consideration for development within the app. Intentional messaging in response to reports and sharing, together with a rotating and novel newsfeed, push notifications, visualization on the map after submission, and appreciation for longer term app engagement can serve as natural and social rewards and positive reinforcement. Incorporating these positive reinforcement elements is designed to bolster user retention, increase reporting of protective actions, and enhance self-efficacy on the user’s path to behavior change and habit formation.

### Reducing friction, bundling benefits.

**Fig. 3:**
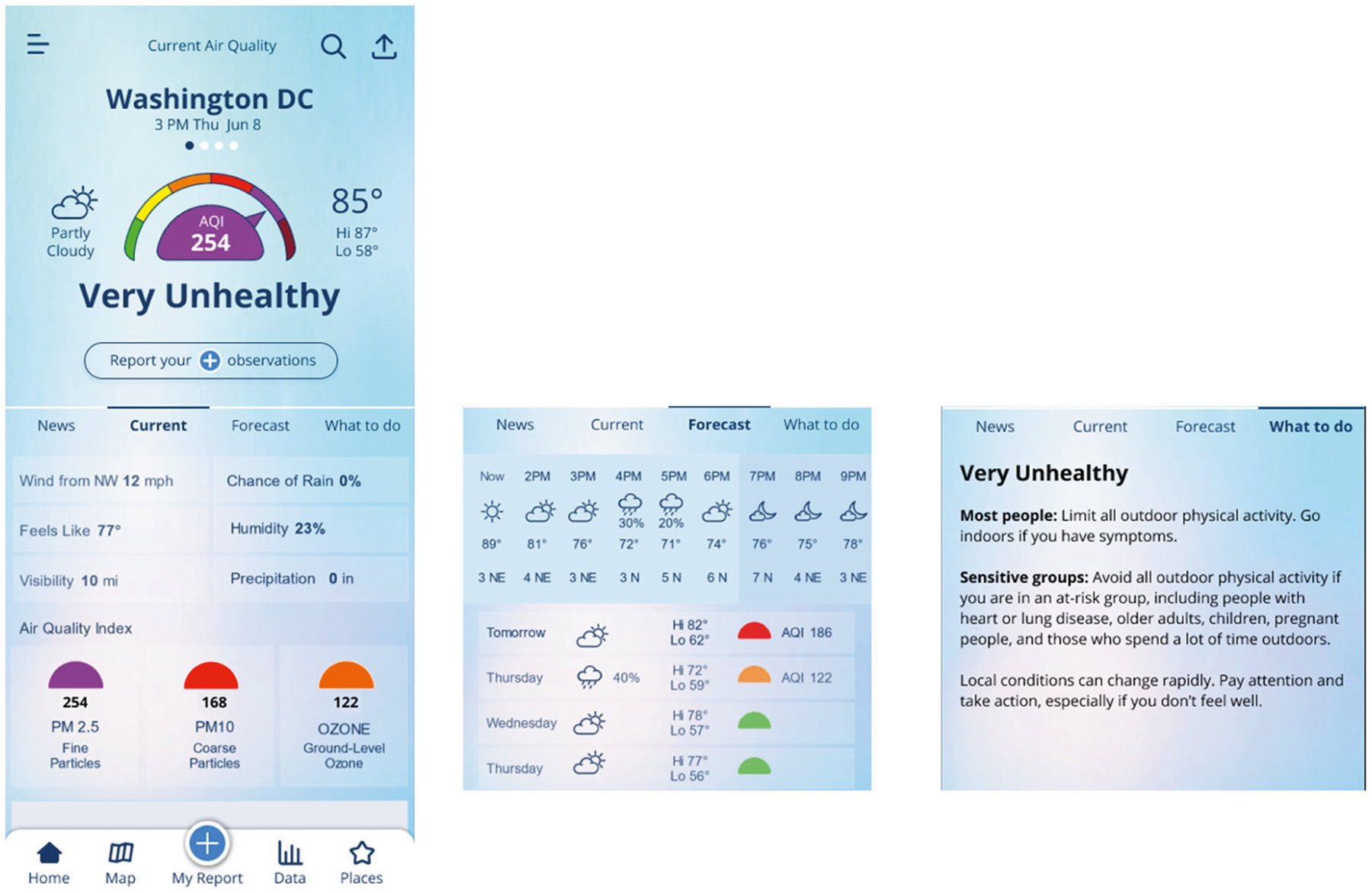
Weather and air quality data. Current weather would be displayed together with air quality data. Forecasts and information about protective behaviors can be presented in the same space to facilitate the connection between conditions and actions.

Barriers or tendencies that make it less likely for a person to take action can be thought of as adding friction. By contrast, steps that make it easier to do something reduce friction associated with the activity (Busso et al., 2015; Irvine et al., 2015). Reward Bundling involves pairing a desirable activity with a less likely performed activity to gain the reward of the former only while engaged in the latter (listening to an audiobook while exercising to increase the likelihood of exercising) (Milkman et al., 2014). By adding current weather information to the user’s home screen (Fig. 3), a perk (and possibly ingrained habit) of checking the weather is bundled with air quality information with each information source also being associated with sets of protective action. Over time, associations between weather conditions and the intensity of fire, smoke, and air quality impacts can become easier for users to understand. This serves the goal of increased awareness but also increased propensity for action. The link between weather and specific actions is often clear, for example a forecast for intense rain signals the need for an umbrella, while one for extreme heat signals the need to tailor activity intensity and outdoor exposure. Thus, the notion of appropriate protective gear and changing behavior to match ambient conditions is a common thread across the default information types presented to the user.

In addition, organizing educational modules into messaging to match the interest or identities of people such as “caregiver of young children,” “outdoor athlete,” or “asthmatics” makes information more salient to the individual and reduces friction associated with sifting through extensive lists. Finally, the app can make it seamless to share information presented with friends, family members, and social networks as a contrast to the tendency to share dramatic images or only the most extreme conditions. The sharing feature within the app can serve to remove friction associated with spreading more basic air quality information and the inherent value of protective actions, while also bundling app usage with the benefit of communicating with friends, family, and others within one’s network. Finally, giving the user community the ability to track up to four unique locations provides an easier path to monitor conditions in the places that are most relevant to their life. Even if a user does not initially intend to take action themselves in their “home location,” the opportunity to learn about community responses in multiple locations can ultimately serve as a guide to best practices and preparedness.

### Self-efficacy.

**Fig. 4:**
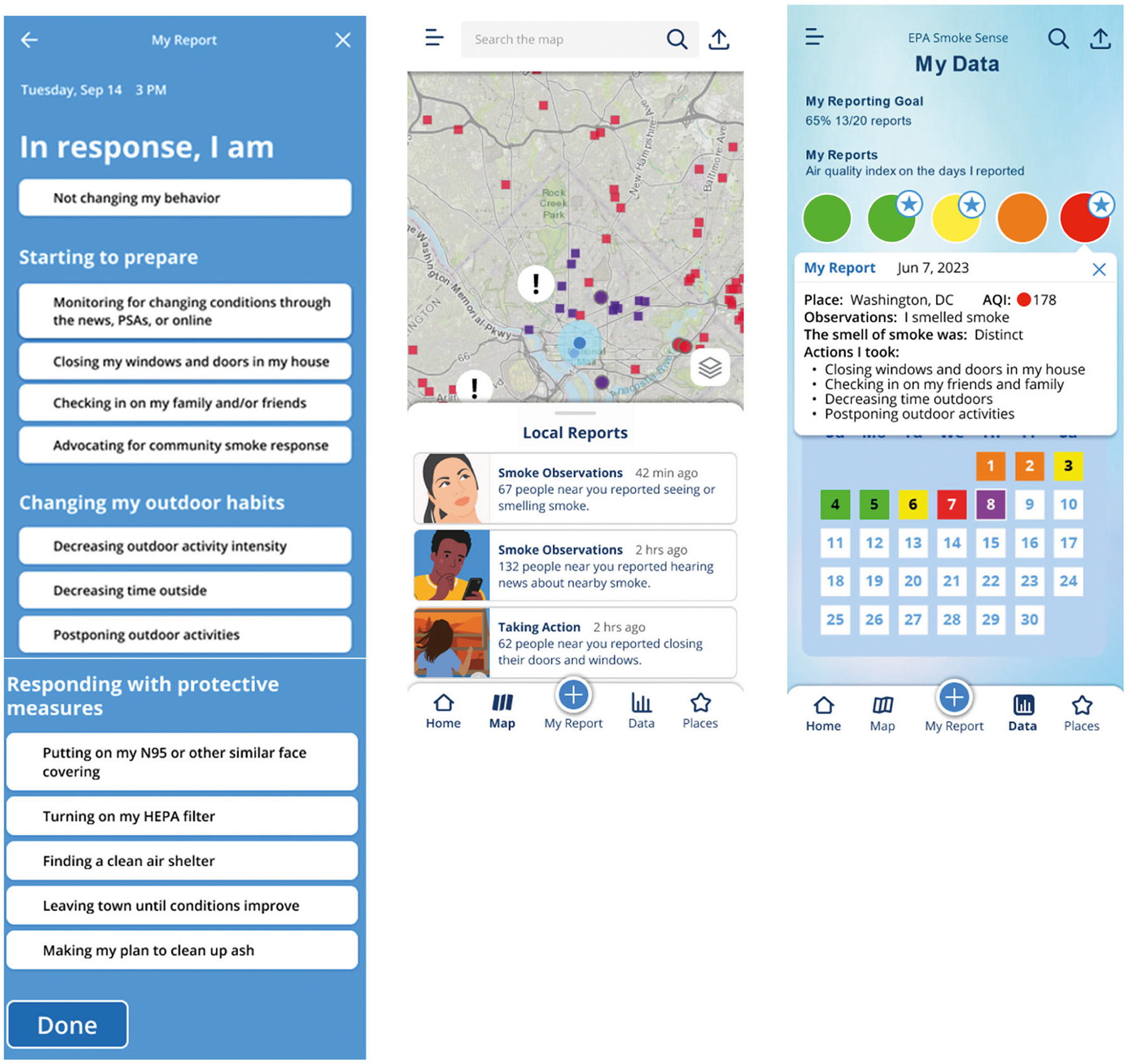
Reports, map view, and my data history. Reports would provide a connection between current conditions and the level of response levels. The map view would provide a summary level for both observations of air quality conditions and actions taken. Reviewing data previously submitted would allow a ‘diary’ or record of conditions and behavioral responses.

Self-efficacy is an individual’s belief in their capacity to act in the ways necessary to reach a specific goal (Bandura, 2004). Social norms, social learning, and social support are all factors that can contribute to increased self-efficacy by providing data about how other people act and feel regarding an issue. As it pertains to air quality and smoke exposure, there is a commonly held idea that smoke is an unavoidable exposure (Santana et al., 2021), or that people take an all or nothing approach to protecting themselves from smoke, when, in fact, there are many small decisions, habits, and protective behaviors people could easily adopt on a day-to-day basis to help protect their health. Giving users the information and tools with which to protect their health will be more useful if it puts recommendations in context as achievable, especially as demonstrated by others. Therefore, messaging and features that instill a sense of self-efficacy can provide users a feeling that tangible outcomes of smoke exposure reduction are realistic and possible (Fig. 4). Establishing a goal to build a record of reports, increases contemplation and self-prediction (how will I respond when air quality conditions change). Such predictions are susceptible to overconfidence, which can benefit from interaction with group data as behavioral norms develop and users continue to contribute (Pronin et al., 2004; Sprott et al., 2006). This record of filed reports is leveraged in the “My Data” feature which allows for reviewing multiple response instances (Kuna et al., 2015), a type of display that lets users reflect on a pattern of successes while not getting overly concerned with a single episode. Although a suite of behavioral responses is available (HEPA filter, mask, windows, reducing indoor pollution sources, exertion level, etc.) users can experience smaller successes as they establish a pattern for deploying specific behaviors.

The goal is for people to feel empowered to protect their health when experiencing smoke exposure. Collectively, social norms, reducing friction, providing reminders and positive feedback, and bundling features can help to change the perception of risks and benefits, and the ease of adopting behaviors. Similar to how social norms facilitate social proof, a user that ‘sees’ others in their community engage and respond with protective behavior might copy that behavior or share information about it with others. When users report responding to remove or lessen particle pollution indoors (using HEPA filter, decreasing frying, and burning candles) the actions gain value as being more achievable. In contrast to passively viewing the information, the contextual data about behavior adoption (when, where, and why) is a ‘boost’ to action. For example, when only images of the orange or darkened skies are shared, they project passive absorption of information and convey a sense of ‘impending doom’ to the receiver. The ability to share health-protective responses is empowering and it challenges the status quo. Ultimately, building resilience in the face of adverse environmental impacts helps us adapt and be less susceptible to harmful health impacts, both as individuals and society at large.

**Fig. 5:**
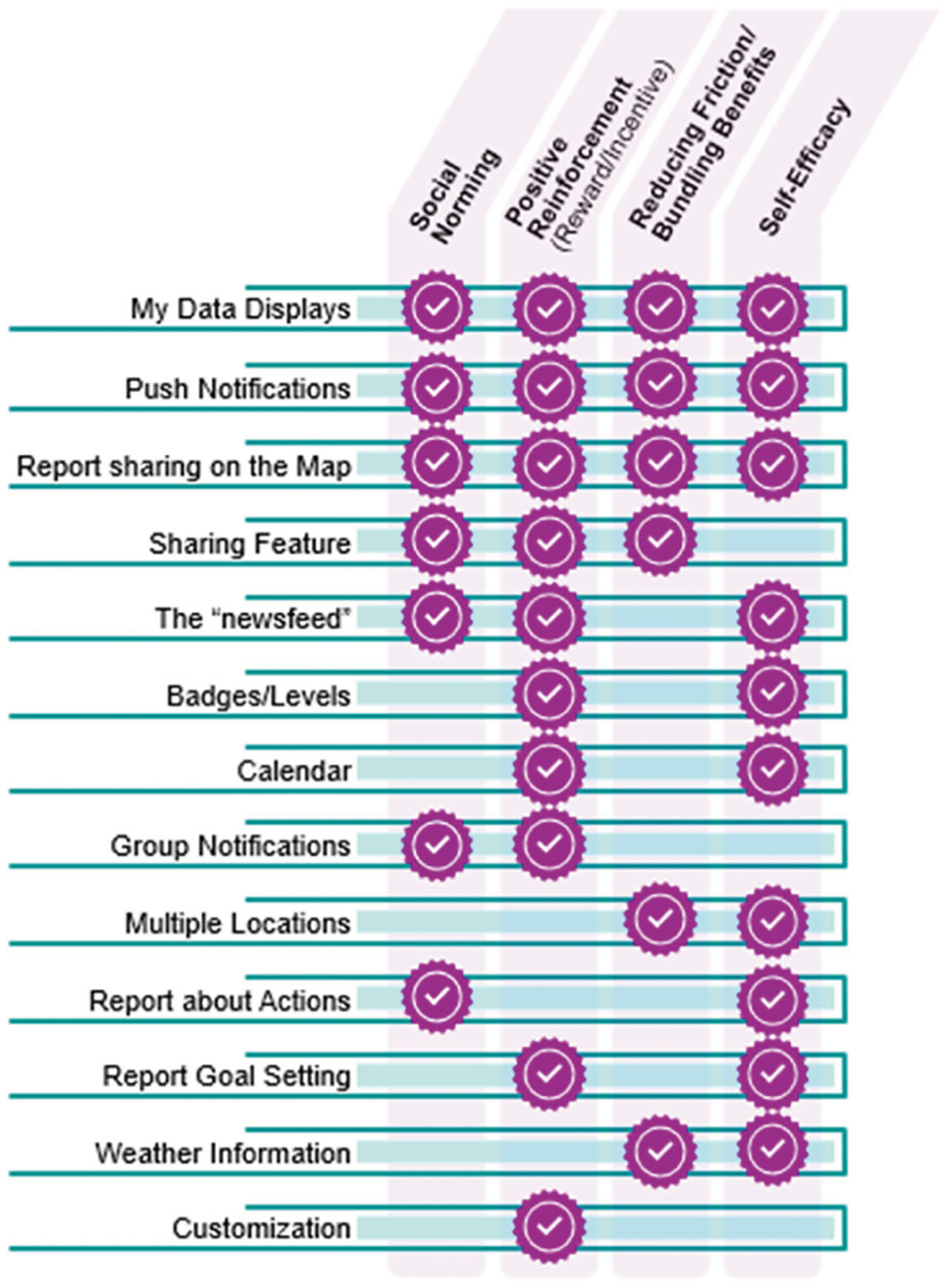
Common social science concepts and where they could be incorporated. Smoke Sense features and interactions would be developed to leverage pillars of behavioral and social science relevant to motivation and habits. The figure is sorted by the intended (number of) pillars a given feature would target.

Based on the insights learned from user inputs, we hypothesize that these common social science concepts- normative learning, reducing friction and bundling benefits, positive reinforcement, and increasing the sense of self efficacy (Fig. 5) can advance participant engagement with environmental health, and more specifically, accepting health protective responses to smoke.

### Preliminary user feedback.

Smoke Sense has offered an interactive and iterative process of learning on the issue of wildfire smoke, perceptions, etc. Based on the data in previous seasons, we translate some of the social science concepts into action making them specific to the issue. New feature concepts were rendered into an interactive mock-up drawing (‘wireframe’). A small group of potential users provided general feedback about the concepts using an interactive wireframe web-based tool. The feedback group sessions were conducted by Sonoma Technology, Inc. (moderated and unmoderated sessions with 15 total participants; 9 female and 6 male ranging in age from 31 to 72; 5 > 60 years of age). Specialized recruiting for moderated sessions yielded 2 participants with asthma and 3 participants who were affected by the Cedar Creek Fire, a wildfire smoke event that resulted in hazardous air quality levels in the town of Oakridge, Oregon in October 2022. Unmoderated sessions were administered to 10 participants recruited through an online platform. Insights from participant sessions pertained to the motivation for how, why, and when the app would be used. Specifically, participating in citizen science research was motivating when descriptions of the real-world data usage were made more salient. Potential users wanted their data to have a clear purpose rather than “sitting” somewhere unknown. People who were actively experiencing poor air quality had specific information needs (how bad are their present conditions, what can they do to immediately reduce harmful impacts) while those with good air quality were most interested in forecasts (planning for the future). As with many popular apps, people reported a preference to check frequently, but briefly, for new information. People preferred ‘useful’ features, but some felt that gamification, competition, or recognition of contributions might enhance motivation for some users. Finally, participants preferred the simplicity of a single pollutant category of the air quality index (PM_2.5_ or fine particle pollution) for the primary display as opposed to multiple pollutants (PM_2.5_ and ozone). These insights were incorporated into design considerations.

### Monitoring and evaluation.

In addition to furthering knowledge about health risk communications and the public response to wildfire smoke events, the Smoke Sense Project seeks to increase users’ motivation to adopt health protective behaviors. The app-based health risk communication can use specific metrics for success to evaluate progress in this area. These metrics include the rates of protective behaviors across all users but focuses more on how individual reports and the propensity to engage with the “My Data” module may change over time. Upon download and initial interaction with the app, users are presented with a default goal, e.g., to complete 10 reports. In previous iterations, reports were typically made on heavy air pollution (smoke) days. By contrast, the proposed features encourage users to report across various air quality conditions they experience to build greater awareness and familiarity with behaviors as they relate to environmental conditions. Providing the anchor value of reports creates a metric that both users and the research team can track. Aggregated statistics based on the number of reports per unit time (day, week, month) can be used to assess how and when users engage with the app. Modifications can be made based on how users interact and whether the initial goal is achievable for most users. Periodic reminders can be delivered to reinforce the role of individual reports in helping to build a community response and greater understanding around smoke exposure and air quality in general.

**Fig. 6:**
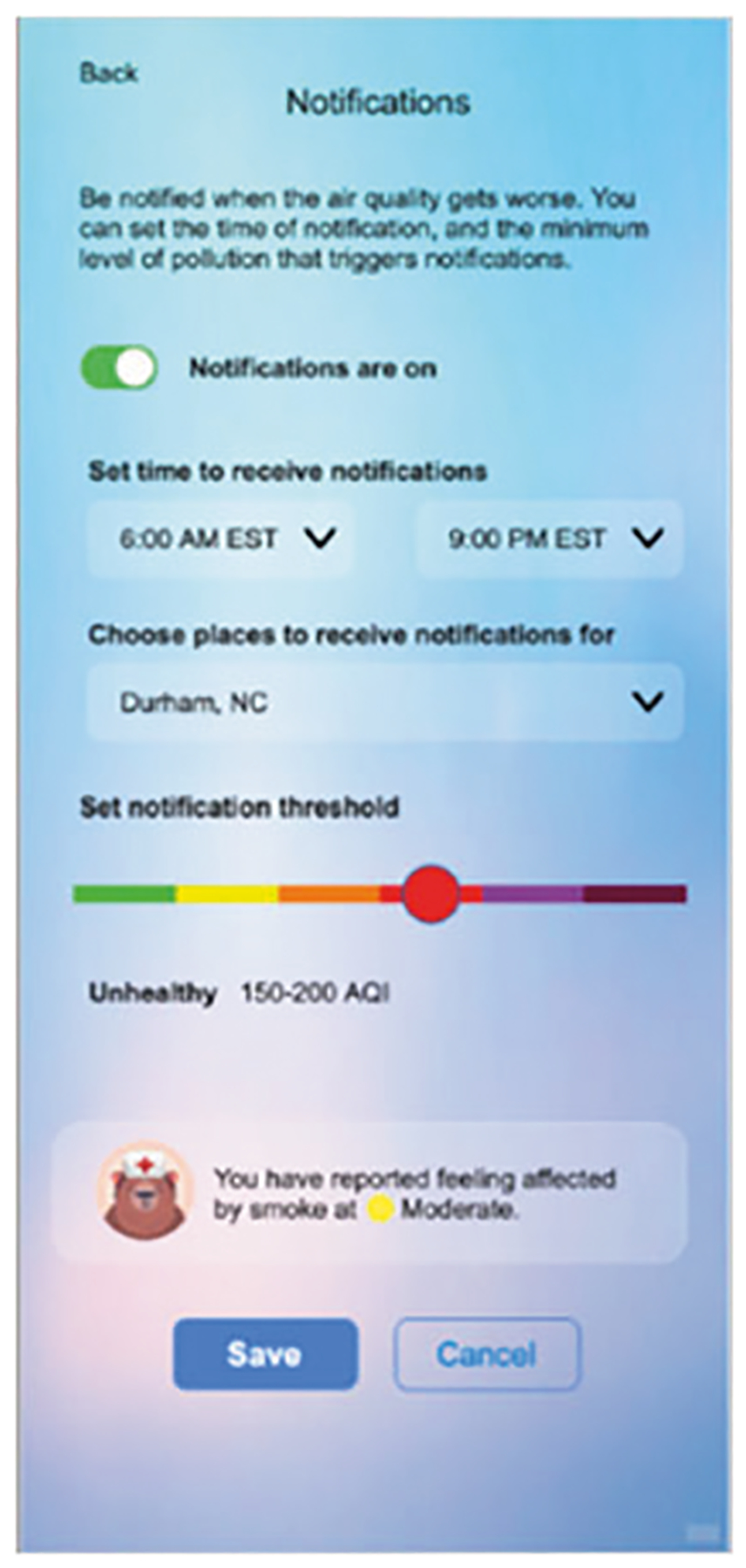
Metrics via push-notifications and settings. User-controlled settings could provide information about air quality importance and whether selections are influenced by location and experience with smoke.

Information gathered about the patterns of use for specific features can be used to evaluate the success of the app and to develop future improvements. For example, these may include frequency of use, time spent on reports, which content is shared, and how user-controlled settings such as push notifications and newsfeed subjects are selected (Fig. 6). In addition, the number of downloads, reports submitted, and Google analytics data regarding session information allow for comparison to previous app versions. A final metric may involve correlating specific external events with downloads or usage of the app. These include the occurrence of large or sustained fires (megafires), media coverage that publicizes the app, and targeted outreach from public health partners. Lessons learned from deploying these new features can be used to help engage people for other difficult topics such as climate change and natural disasters (e.g., flood preparation, hurricane evacuation and sheltering from tornadoes). Finally, we plan to further engage users who reach their reporting goals to participate in selecting improvements and new features for the app. This broader app development process of monitoring, evaluating, and iterating has the potential to better engage and motivate users, while providing timely and actionable information in a commonly accessible platform.

## Discussion

### Looking out to the horizon.

The issue and impact of wildfire smoke exposure has a massive scope in the United States that will only increase given demographic trends of wildland urban interface population growth and the frequency of extreme drought and heat conditions driven by climate change. Catastrophic wildfires in the Western U.S. over the last decade have been the impetus for the growing body of research on wildland fire smoke around such topics as the intersection of climate and wildfire as well as the potential public health implications of poor air quality due to smoke, with federal responses to the wildfire crisis including an Executive Order signed by President Biden to Strengthen America’s Forests, Boost Wildfire Resilience, and Combat Global Deforestation.

Responding to this issue will require new and innovative approaches to engage and motivate people who have varied levels of interest, expertise, and familiarity with getting and understanding the relevant information and taking action to reduce exposure. Providing air quality information is but one prong of a multi-faceted approach to engage with the complexity and uncertainty brought about by wildfires. Information needs to be available and accessible at the time and place it is most needed, with language and contextual details that convey the importance and urgency of action. The social science tools described herein constitute a framework to guide users through a learning process. To keep individual users engaged, features such as app customization, notifications, and eliciting feedback from committed users will supplement and allow for further iterative feature enhancement. Individual citizen scientists who interact with Smoke Sense create a community in which others can learn. Over time, these efforts can familiarize users with navigating air quality patterns and the impacts of wildfire smoke exposure. Individuals with shared geography who face similar environmental threats can become a network for social support; a ‘digital community’ that reports on conditions and actions helps to create a broader informed community. Familiarity and preparedness help to reduce fear and uncertainty while motivating actions toward a common health protective goal. In other words, with smoke more frequently in the air, the user community gains a sense of what is on the horizon, together with a sense of how to respond.

Citizen science has long been used as a tool to collect broader reaching data and actively engage the public with knowledge gathering. Citizen science and crowdsourcing are also referred to as tools for democratization of knowledge because they simultaneously engage and educate the public through voluntary engagement on the issue that interests them. Citizen Scientists contribute to open collaboration by actively participating in various stages of the scientific process (defining research questions, collecting, and analyzing data, interpreting results, or engaging in problem solving). Citizen science can be particularly effective when the issue affects daily life. Scientific projects specifically related to natural disasters provide several examples of citizen science approaches that have improved public understanding and emergency preparedness for dangerous weather events and natural phenomenon (Riesch and Potter, 2014). Smartphone applications are an effective method for citizen science projects to reach audiences when and where they are experiencing an issue. Several projects have utilized smartphone applications, including CoCoRaHS, iNaturalist, ISeeChange, and MyShake. These apps cater to topics ranging from precipitation measurement to biodiversity and climate change observation, to earthquake detection, characterization, and protective actions.

## Conclusion

Environmental disasters are an increasingly common experience that force communities to strengthen their resilience while they are responding to crisis. Responding to environmental disasters requires a multifaceted approach and the ability to reach the affected individuals during changing and complex conditions. However, in most environmental instances, there is no single agency, organization, or entity that can respond with a solution that addresses everyone’s needs. In the United States, President Obama’s Administration issued the 2013 Second Open Government National Action Plan calling on agencies to leverage the ingenuity of the public through the use of citizen science and crowdsourcing to provide timely and actionable information (Holdren, 2015). This memorandum provides an impetus to utilize citizen science approaches to “enhance scientific research and address societal needs, while drawing on previously underutilized resources” and information available to government. Most importantly, citizen science participation allows individuals from all walks of life to engage on an issue and expand knowledge for the benefit of the community. Smoke Sense is a citizen science research platform for iteratively building knowledge about wildfire smoke, health, and protective actions rooted in epidemiologic, clinical, and social science. Taking an iterative approach enabled problem formulation to be crowdsourced across 60,000+ citizen scientists who provided their context of the wildfire smoke experience. In this manuscript, we now propose a “level up” iteration in which common pillars of behavioral science theory are used to motivate engagement, participation, and sharing of knowledge to meet societal needs, build resilience, and improve public health responses. Finally, the concept introduced here can be used more widely to learn and implement health risk communication strategies that address the context and experience of users during environmental disasters.

## Figures and Tables

**Table 1:** Potential barriers to action related to wildfire smoke exposure.

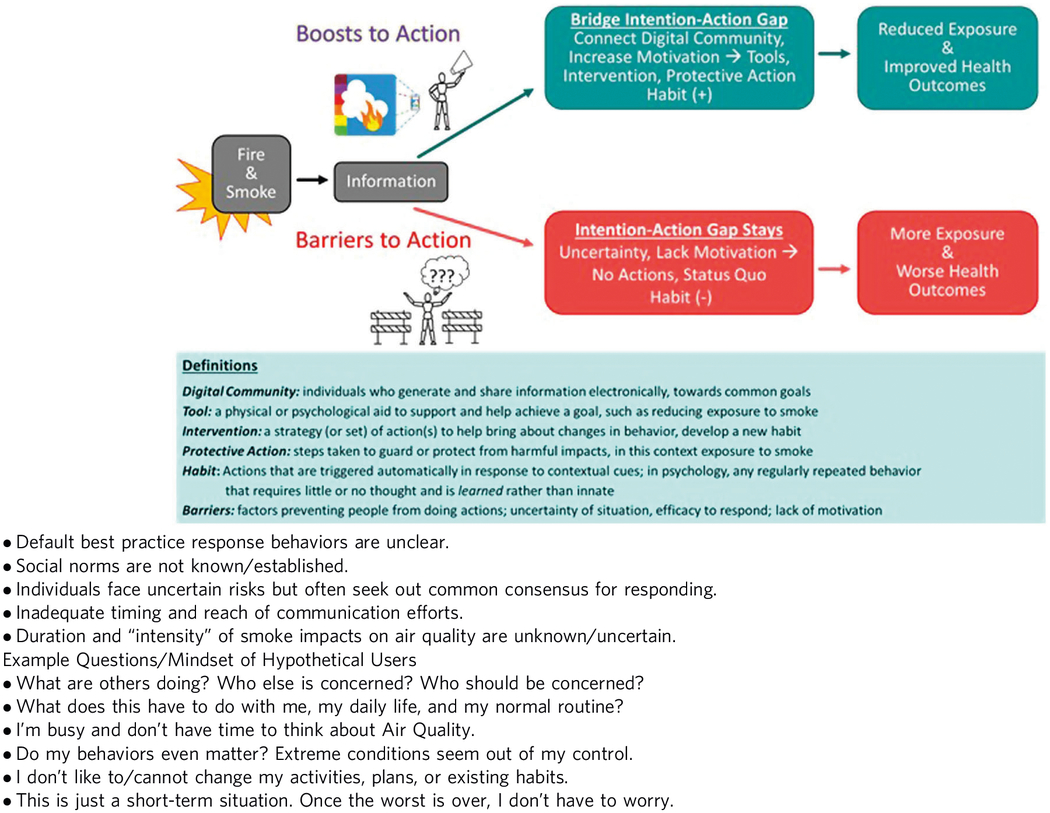

## Data Availability

Data sharing is not applicable to this research as no data were generated or analyzed.
